# Incidental detection of left ventricular metastasis from lung squamous cell carcinoma on FDG PET/CT: A rare case report

**DOI:** 10.22038/aojnmb.2026.92391.1682

**Published:** 2026

**Authors:** Marwah Abdulrahman, Ahmed Abdlkadir, Nouraldeen Al-zorgan, Bara'a Bashabsheh, Akram Al-Ibraheem

**Affiliations:** 1 Department of Nuclear Medicine, King Hussein Cancer Center (KHCC), Amman, Jordan; 2 School of Medicine, the University of Jordan, Amman, Jordan

**Keywords:** Lung cancer, Squamous cell carcinoma, FDG PET/CT, Cardiac metastasis, Left Ventricular metastasis

## Abstract

Cardiac metastases are uncommon, particularly in the left ventricle, where lesions often remain asymptomatic and can be easily overlooked on conventional imaging. Lung cancer, a leading cause of global cancer-related morbidity and mortality, accounts for approximately one-third of all cases of cardiac metastases among various malignancies. We present a 62-year-old male with stage IV squamous cell carcinoma of the lung, who exhibited widespread systemic metastases. Staging Fluorine-18 Fluorodeoxyglucose (¹⁸F-FDG) Positron Emission Tomography/Computed Tomography (PET/CT) revealed a hypermetabolic cavitary left upper lobe mass with a metastatic process involving multiple nodal, adrenal, hepatic, osseous, and cerebral deposits, along with an incidental focal FDG-avid lesion in the left ventricle that was not detected on initial CT. Echocardiography confirmed preserved cardiac function with a normal ejection fraction. Given uncommon nature of left ventricular involvement by squamous cell carcinoma and the limited number of similar reported cases, our findings contribute to the growing evidence supporting the critical role of ¹⁸F-FDG PET/CT in detecting clinically silent occult cardiac metastasis.

## Introduction

 Lung cancer remains the first leading cause of cancer-related morbidity and mortality worldwide(1). It constitutes about one-eighth of all new cancer diagnoses and nearly one-fifth of global cancer deaths, ranking first in incidence and mortality among men and second among women(1). Although adenocarcinoma is now the most common histologic subtype, squamous cell carcinoma continues to represent a substantial proportion, accounting for roughly 30% of all non-small cell lung cancer (NSCLC) cases and being especially common among long-term smokers(2). The high mortality of lung cancer is driven by its propensity for early metastatic spread(3). The most common documented metastatic sites in NSCLC include the brain, bone, adrenal glands, and contralateral lung(4). However, cardiac metastasis remains a rare occurrence, with involvement of the left ventricle being even less commonly reported. Notably, lung cancer accounts for approximately one-third of all cases of cardiac metastases among various malignancies(5).

 Although cardiac metastases from NSCLC, particularly squamous cell carcinoma, have been documented in the literature(6,7), reports specifically addressing left ventricular metastases remain limited(8),(9). The role of Fluorine-18 Fluorodeoxyglucose (¹⁸F-FDG) Positron Emission Tomography/Computed Tomography (PET/CT) in evaluating cardiac masses is evolving, as it serves as a complementary modality for distinguishing benign from malignant

lesions based on metabolic activity while also supporting lung cancer staging and restaging by detecting both overt and occult metastatic disease(8),(10). Beyond its diagnostic value, ^18^F-FDG PET/CT provides crucial metabolic information that often reveals unexpected metastatic sites not apparent on anatomical imaging(11).

 Herein, we report a distinctive finding of left pulmonary squamous cell carcinoma, characterized by a widespread metastatic process affecting multiple sites, including the infrequent site of the left ventricle. Malignancies may metastasize to cardiac tissue, but diagnostic uncertainty and possible false-positive ¹⁸F-FDG uptake make multimodality imaging important for accurate characterization of intracardiac lesions(12).

## Case presentation

 A 62-year-old male was referred to our hospital with a primary complaint of chronic productive cough that had progressively worsened over several weeks. The cough was occasionally accompanied by hemoptysis, along with intermittent episodes of shortness of breath and unexplained weight loss of approximately 10 pounds over the previous six months. The patient reported a significant history of heavy smoking, averaging three packs per day for the past five decades. His family history included leukemia and pancreatic cancer, but his personal past medical history was otherwise unremarkable. 

 Diagnostic workup included initial imaging with a chest X-ray (CXR) which revealed a large mass in the left upper lobe. To further characterize the lesion, contrast enhanced computed tomography (CECT) of neck, chest and abdomen was performed, showing a spiculated left hilar and left upper lobe’s lateral aspect soft tissue density mass with multiple cavitary areas, surrounded by diffuse pulmonary infiltrates, In addition to multiple pulmonary and pleural-based nodules, prominent mediastinal lymphadenopathy with necrosis, and no pleural effusion. The abdomen and pelvis showed bilateral adrenal masses and para-aortic, mesenteric, and omental soft tissue deposits. Furthermore, brain magnetic resonance imaging (MRI) confirmed the presence of two enhancing lesions in the right frontal lobe likely representing secondary deposits. 

 This initial presentation prompted the diagnosis of malignant lung tumor which was verified by histopathological findings of mediastinal lymph node biopsy revealing metastatic poorly differentiated squamous cell carcinoma. Immunohistochemistry revealed Thyroid Transcription Factor-1 (TTF-1) positivity, focal P40 positivity, Anaplastic Lymphoma Kinase (ALK) negativity, and Programmed Death-Ligand 1 (PD-L1) positivity with a Tumor Proportion Score (TPS) of 25%. Among serological tests, the results were negative for Hepatitis B Core Antibody (Total), Hepatitis C Virus Antibody, Hepatitis B Surface Antibody, and Hepatitis B Surface Antigen.

**Figure 1:**
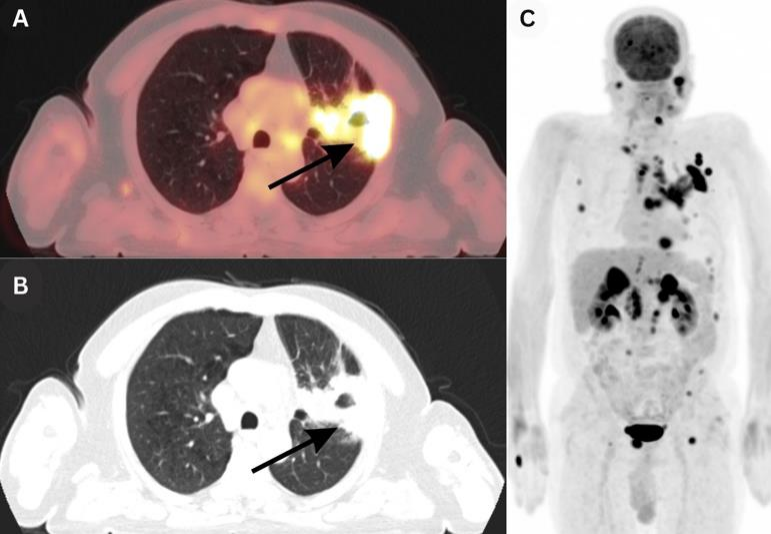
Fused (**A**) and CT (**B**) images of ^18^F-FDG PET/CT scan on axial plane, lung window showing a large spiculated soft tissue mass in the left upper lobe, with cavitary component (**black arrows**), this mass is associated with consolidative changes and pleural tethering, infiltrating the left 3rd and 4th ribs. The mass is exhibiting an intense FDG uptake (SUV_max_: 20.7), and measuring collectively with the consolidative part of it (5.1×7.4 cm) in maximal axial dimensions. Maximum intensity projection (MIP) demonstrating hypermetabolic widespread metastases (**C**)

**Figure 2:**
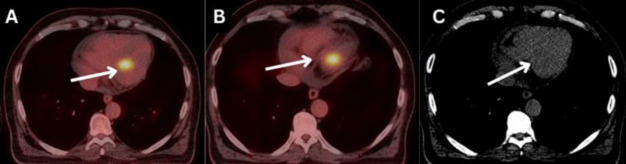
^18^F-FDG PET/CT scan, axial planes of fused images (**A** & **B**) showing an intense focus of FDG activity (SUV_max_:10.8) within the left ventricle (**white arrows**) measuring about 0.4×0.3 cm in maximal active component which correlate to a soft tissue density mass on corresponding CT component (**C**) (**white arrow**)

**Figure 3:**
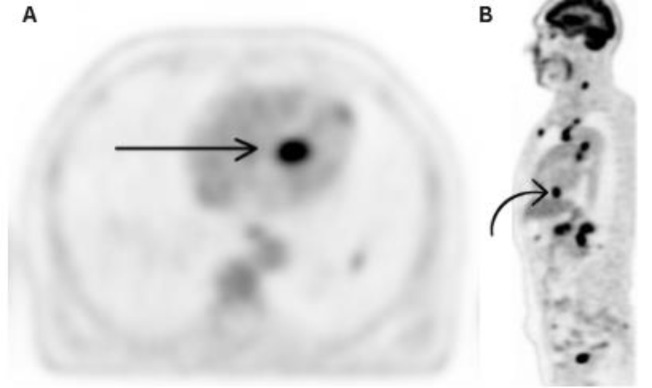
**.** ^18^F-FDG PET scan axial (**A**) and sagittal (**B**) views demonstrating the cardiac left intraventricular focal metastatic lesion (**black arrow** and **curved arrow** respectively)

 Subsequent ^18^F-FDG PET/CT imaging demonstrated a hypermetabolic malignant cavitary mass in the left lung, consistent with the biopsy-proven primary tumor. Extensive hypermetabolic metastatic process involving the supraclavicular, mediastinal, axillary, abdominal, and inguinal lymph nodes, hypermetabolic bilateral intraparotid nodules, bilateral pulmonary nodules, bilateral adrenal lesions, liver, multiple peritoneal and omental deposits, multiple osseous lesions, a suprapubic subcutaneous nodular deposit and a right frontal lobe brain lesion. (Figure 1) Interestingly, a focal FDG-avid lesion within the left intraventricular cardiac region was missed on CECT, warranting dedicated cardiac imaging for further evaluation (Figure 2, 3). 

 However, the echocardiographic study demonstrated normal cardiac anatomy and preserved systolic function, with a left ventricular ejection fraction (EF) of 50–55%. No wall motion abnormalities, valvular dysfunction, or intracardiac masses were observed, supporting the incidental nature of the left ventricular lesion detected on PET/CT. Cardiac MRI was not performed, as the lesion was small, asymptomatic, and adequately characterized by PET/CT and echocardiography.

 The multidisciplinary team (MDT) recommended initiating a chemotherapy regimen consisting of six cycles of paclitaxel and carboplatin, to be followed by a subsequent PET scan for evaluation. The patient has completed the first cycle with good tolerance.

## Discussion

 Multisystemic metastatic involvement is frequent in NSCLC, with around 65% of patients identified at an advanced stage, reflecting its aggressive biological behavior and early hematogenous dissemination(13). In this context, ^18^F-FDG PET/CT plays a pivotal role, not only in detecting both overt and occult metastases across multiple organ systems but also in refining staging, guiding therapeutic strategies, and monitoring treatment response(14).

 Despite remarkable advances in diagnostics and therapy, the overall 5-year relative survival rate for NSCLC remains around 28%(15). Survival rates vary significantly by stage at diagnosis, with localized NSCLC having a 5-year relative survival rate of 65%, regional at 37%, and distant at 9%(15), reflecting the significance of earlier and accurate staging for improving long-term outcomes and overall survival rates.

 Cardiac metastasis is often overlooked, as many cases remain asymptomatic. Studies showed that cardiac metastases have been identified in up to 25% of patients with malignancies during autopsy examinations(16). Dissemination from malignant tumors may reach the heart through four primary mechanisms: hematogenous dissemination, lymphatic invasion, transvenous migration, and direct tissue infiltration. The pericardium and epicardium are the most frequent sites of involvement, with lymphatic spread being the predominant route of metastasis in such cases(17).

 Conventional imaging modalities, such as transthoracic echocardiography (2D and 3D) and CT, are typically guided by clinical symptoms like dyspnea, syncope, or ECG abnormalities. ^18^F-FDG PET/CT on the contrary, can incidentally identify cardiac metastases during routine oncologic staging, detecting metabolically active lesions that may remain clinically silent and thereby improving diagnostic accuracy(18). Nonetheless, the identification of cardiac lesions using ^18^F-FDG PET/CT can be challenging under standard preparation protocols due to the high physiological FDG uptake in the myocardium and its significant variability(19). Yet despite these limitations, any focal myocardial FDG uptake should be inspected meticulously for potential cardiac metastases, which have been documented in 1.5% to 20% of cancer patient autopsies(20). 

 Focal ¹⁸F-FDG uptake in the left ventricular myocardium is not specific for metastatic involvement and warrants careful differential consideration. Benign entities such as thrombus, lipoma, papillary fibroelastoma, inflammatory lesions, or even physiologic myocardial uptake—particularly in cases of suboptimal metabolic preparation—may mimic malignancy. Prior studies have demonstrated that malignant cardiac and pericardial lesions exhibit significantly higher metabolic activity than benign masses with reported median SUV_max_ values of 6.5 for malignant lesions compared with 1.5 for benign entities (21). 

 Similarly, Qin et al. showed that SUV_max_ provides strong diagnostic performance in differentiating benign from malignant cardiac masses and carries independent prognostic significance, with a SUV_max_ cutoff of approximately 6.7 yielding high sensitivity (92.1%) and specificity (88.9%). The study also emphasized the added value of integrating metabolic indices with CT morphological features, as combined assessment improved diagnostic accuracy(22).

 A few studies on myocardial localization of NSCLC metastasis have been published in literature to date. Tagliabue et al. were the first to report such instance, with a relapsed acinar adenocarcinoma case with myocardial metastasis in the ventricular septum, which was exclusively detected by a follow-up ^18^F-FDG PET/CT(23). Similarly, case reports described intramyocardial implantation, with lesions identified in structures such as the interventricular septum and ventricular walls, often detected incidentally during staging with contrast-enhanced transthoracic echocardio-graphy and cardiac CT(7)^, ^or functional imaging with ^18^F-FDG PET/CT⁷. The clinical significance of such metastases is substantial, as they are not always silent. Notably, left ventricular involvement carries a risk of major complications, including myocardial infarction through the occlusion of the coronary artery by a metastatic mass originating from squamous cell lung cancer(16). A recently reported case of stage IV lung squamous cell carcinoma with cardiac metastases highlights the combined utility of echocardiography and ^18^F-FDG-PET/CT for diagnosis. The authors notably observed a marked response to immunotherapy in a tumor with high PD-L1 expression, resulting in the regression of both the primary lesion and cardiac deposits(8).

 This case highlights that ¹⁸F-FDG PET/CT may incidentally detect clinically silent left ventricular metastasis during routine oncologic staging. While PET/CT provides valuable metabolic information that can raise suspicion for malignant cardiac involvement, its findings should be interpreted within a multimodality imaging framework, integrating echocardio-graphy, cardiac CT, or cardiac magnetic resonance when appropriate(24). 
